# The Ophthalmic Side Effects of Topiramate: A Review

**DOI:** 10.7759/cureus.28513

**Published:** 2022-08-28

**Authors:** Monia Mechrgui, Suleman Kanani

**Affiliations:** 1 Ophthalmology, Primary Health Care Corporation (PHCC), Doha, QAT; 2 Internal Medicine, Primary Health Care Corporation (PHCC), Doha, QAT

**Keywords:** ciliochoroidal effusion syndrome, induced myopia, angle closure glaucoma, ocular side effects, epilepsy

## Abstract

Topiramate (TPM) is a sulfonamide drug with multiple modes of action. It inhibits carbonic anhydrase, blocks sodium channels, enhances potassium channels, and stimulates postsynaptic gamma-aminobutyric acid (GABA) receptors. Pharmacists Joe Gardocki and Bruce Maryanoff synthesized TPM for the first time in 1979. The FDA did not approve it for medical use in the US until 1996. Around 2004, it was authorized for the prevention of migraine headaches.

TPM, like any medication, has several side effects. Common aftermaths include weight loss, diarrhea, dizziness, sleepiness, fatigue, and coordination issues. Some people may experience mental health issues like memory problems, confusion, and speech or language difficulties. The most well-known ocular side effects of TPM are choroidal effusion syndrome, angle-closure glaucoma, and myopic shift. Aside from these, other ophthalmic adverse effects may arise in some people, including retinal problems, uveitis, visual field defects, myokymia, and neuro-ophthalmology complications. If such complications are not identified and treated promptly, they can be severe and vision-threatening, potentially leading to permanent blindness.

TPM's application as a standalone and adjunctive therapy has increased over time. In 2019, more than 10 million prescriptions of TPM were issued. Due to its extensive use, medical professionals and patients must be aware of its potential repercussions, especially ophthalmic issues. The current review paper likewise makes a step in this direction. This article's primary purpose is to educate readers by providing a comprehensive assessment of the research on TPM's ocular side effects. All the information has been collected via a thorough search of the Google Search Engine and PubMed.

## Introduction and background

Topiramate (TPM) is a sulfamate-substituted drug with multiple mechanisms of action. It inhibits sodium channels while enhancing potassium channels, blocks carbonic anhydrase, and stimulates postsynaptic gamma-aminobutyric acid (GABA) receptors [1]. TPM is excreted through the kidneys and has an elimination half-life of approximately 24 hours [2].

TPM was first synthesized by pharmacists Joe Gardocki and Bruce Maryanoff in 1979 during their research at Ortho-McNeil Pharmaceuticals. When researchers tested it on mice, its anticonvulsant activity became apparent. The US FDA initially approved TPM as an antiepileptic medicine for adjunctive therapy with other agents in 1996 [3]. In 2004, it was approved to deter migraine headaches in adults. Furthermore, the FDA permitted the extended-release formulation in combination with phentermine for adult weight management in 2012 [4]. TPM is currently used for migraine prevention and partial-onset and generalized tonic-clonic seizures in adolescents and adults 12 years of age and older [5]. Off-label uses of TPM include bipolar disorder, borderline personality disorder, alcoholism, and binge eating disorder. In 2019, TPM was the 68th most frequently prescribed medication, with over 10 million prescriptions written [6].

As with any medication, TPM comes with a list of side effects. Fatigue, sleepiness, dizziness, uncoordinated movements, paresthesias, dysgeusia, diarrhea, and weight loss may also happen. Mental issues such as confusion, difficulty concentrating, memory issues, and problems with speech/language may also occur [7]. The most well-known ocular side effect is the choroidal effusion syndrome, which may lead to acute angle-closure glaucoma (AACG) and a dramatic myopic shift. Other issues may include retinal complications, uveitis, visual field defects, myokymia, neuro-ophthalmic complications, increased corneal thickness, and possibly scleritis. The risk of an adverse reaction to any sulfonamide drug is approximately 3% [8]. The overall rate of ocular side effects induced by TPM is not known. However, the rate of the most dreaded ocular complication, AACG, has been estimated to be approximately three out of 100,000 [9].

TPM's indications and usage have expanded over time as a standalone and adjunctive therapy. However, with millions of prescriptions each year, medical care providers and patients must be aware of the consequences, particularly ophthalmic complications. It can be severe and vision-threatening, potentially leading to permanent blindness if they are not recognized and managed urgently. This article aims to provide a comprehensive review of TPM's ocular side effects for educational purposes.

## Review

Methods

The information included in this review was collected through a detailed search on Google Search Engine and PubMed. Retrospective literature reviews, prospective studies, and case studies were all taken into consideration. To provide useful information in a single review article, results were collected and combined in a coherent, well-ordered manner.

TPM ophthalmic complications

Ciliochoroidal Effusion Syndrome

A ciliochoroidal effusion is an abnormal accumulation of fluid in the suprachoroidal space of the eye that can cause a forward rotation of the ciliary body and forward movement of the lens-iris diaphragm (LID). In some cases, this can lead to a transient myopic shift in vision or even blockage of the eye's internal drainage system, leading to secondary AACG. Ciliochoroidal effusion can be caused by glaucoma surgery, trauma, inflammation, infection, neoplasm, or a medication side effect. The mechanism by which TPM may cause ciliochoroidal effusions is not well understood. Ciliochoroidal effusion can develop days or weeks after starting TPM [10].

Acute Angle-Closure Glaucoma (AACG)

The major complication of ciliochoroidal effusion syndrome is secondary angle-closure glaucoma (ACG). TPM-induced angle-closure glaucoma (TiACG) is a bilateral ocular condition. It results in ocular pain and headache that typically occurs for minutes to hours, accompanied by nausea or vomiting. In addition, the slit lamp examination may reveal a flat or a shallow anterior chamber and inflammation, corneal edema, and conjunctival injection. 

Early diagnosis and discontinuation of the offending medication are critical in treating TiACG. Thus, patients using TPM should be informed about symptoms of angle-closure beforehand and encouraged to seek immediate medical help if any symptoms appear. Typically, surgical intervention is not required in this condition. Peripheral iridotomy and topical miotics are ineffective because the angle-closure mechanism is not pupillary block in these cases [11]. Likewise, prostaglandins are contraindicated given the anatomical obstruction of the uveoscleral outflow system in angle-closure and their potential to instigate inflammation.

Topical and oral eye pressure-lowering medicines along with mydriatic eye drops (atropine) are recommended. In addition, steroid eye drops may be helpful due to the comorbid inflammation. Acetazolamide, another sulfa-derived drug, should be used cautiously as it may exacerbate the problem [12]. In severe or refractory cases, systemic steroids and hyperosmotic agents such as mannitol may be considered [13].

As early as 2001, Banta JT et al. presented a case of bilateral ACG associated with oral TPM therapy. In this case, a 51-year-old man developed this condition two weeks after starting TPM therapy for bipolar affective disorder. On his right eye, the laser peripheral iridotomy was performed without resolution. B-scan USG revealed ciliochoroidal detachments and lens thickening in both eyes. Two weeks after discontinuing TPM therapy, the patient's anterior and posterior segment anatomy, intraocular pressure (IOP), and visual acuity became normal [13]. The manufacturer and distributor of TOPAMAX®, Ortho-McNeil Pharmaceuticals, issued a statement in 2001 stating that 21 cases of AACG had been reported to its Safety Division. There have also been reports of TPM-associated glaucoma detected by high-frequency ultrasound, revealing forward displacement of the LID and ciliary process swelling [14]. In 2004, Craig JE et al. concluded that TPM might be associated with choroidal effusion with anterior chamber shallowing and LID's forward displacement. It can lead to ACG and acute myopia. Increased lens thickness had a minor impact on the anterior chamber shallowing [15].

Overall, TiACG is rare. Compared to primary angle-closure glaucoma, this condition is usually bilateral and affects young people (less than 40 years of age). This condition has been reported to occur more commonly in female patients, but this difference may be explained by the fact that women use the medication more frequently [9].

Although the mechanism of TiACG is not well-known, one theory proposes that this drug may cause excessive fluid accumulation within the ciliary body and choroid. It is due to the increased permeability of the ciliochoroidal vasculature. The iris-lens diaphragm is pushed forward by edema and subsequent choroid thickening, resulting in increased retro-lenticular pressure [16]. Ciliary body swelling causes the anterior rotation of the ciliary processes, further exacerbating forward displacement of the LID, leading to angle-closure. Furthermore, ciliary body swelling reduces zonular tension, leading to an increase in lens thickness. This displacement of the iris and lens instigates the commonly reported myopic shift. Furthermore, inflammation may play a role as numerous reports of uveitis-related cases exist in the literature. There are also reports of refractory cases in the literature that responded well to high-dose steroids [17].

The prognosis is favorable if the medicine is discontinued early and proper treatment is provided. IOP, refractive error, and visual acuity usually return to normal as the ciliochoroidal effusions resolve. However, if the condition is not properly diagnosed, persistent high IOP can cause permanent damage to the optic nerve [18].

Myopic Shift

In addition to AACG caused by the forward rotation of the LID and the ciliary body edema, patients taking TPM may experience a significant myopic shift. This myopic shift may not induce AACG. They are treated similarly, with cessation of TPM and the use of cycloplegic agents (for example, atropine) to expand the anterior chamber and reverse the rotation of the ciliary body. TPM may induce several diopters of myopia. The myopic shift is variable, with a range of -2.00 to -8.75 diopters, likely correlating to the displacement of the crystalline lens [19]. Resolution of the myopic shift typically occurs with the cessation of TPM therapy. Patients should be observed with serial IOP checks in the ensuing days to ensure they do not develop angle-closure. Mydriatics and oral steroids have sometimes been used to speed resolution [20].

Retinal complications

Retinal Striae

The association between retinal striae and TPM use has been previously reported [21]. This is attributed to posterior choroidal effusion. Cases of retinal folds usually report a myopic shift secondary to the anterior displacement of the lens. The displacement shifts the anterior hyaloid forward and widens the anteroposterior diameter of the vitreous cortex in a setting of a taut posterior hyaloid. This shift may cause retinal traction that induces folds at the internal limiting membrane (ILM) level. Upon clinical examination, this traction would appear as superficial striae [22]. TPM's discontinuation resulted in resolution of retinal striae and rapid improvement in visual acuity. 

Maculopathy With Neurosensory Retinal Detachment

In addition to retinal striae, TPM may cause macular and retinal neurosensory detachments. Symptoms improve once TPM is discontinued [23-24]. In 2012, Gualtieri W and Janula J reported the first case of TPM-induced maculopathy, citing a report of a 22-year-old female who developed a neurosensory retinal detachment and macular striae six days after beginning TPM for a migraine attack. The retinal detachment and striae resolved after the discontinuation of TPM and the use of steroids [25]. Two other articles have since been published describing this phenomenon [23-26].

Uveitis

The propensity for TPM to induce ocular inflammation is less well-known than other side effects. However, it can cause severe anterior uveitis with hypopyon formation. Few reports have associated TPM with anterior uveitis, hypopyon uveitis, and panuveitis [27-30]. Hypotony can sometimes occur with this inflammation and is attributed to the ciliary body shutting down and then related to choroidal detachments caused by the inflammation. Treatment consists of topical and often systemic steroids. TPM-induced uveitis is considered very rare. There is currently no evidence of duration or dose-dependent relationship between TPM use and uveitis [28].

Visual Field Defects

Visual field defects secondary to TPM are commonly attributed to angle-closure glaucoma due to ciliochoroidal effusion syndrome. In such cases, the visual field abnormalities are frequently accompanied by pain because of increased IOP. Additionally, TPM users reportedly experience visual scotomas due to maculopathy and retinal damage. Therefore, when patients are being considered for TPM therapy, baseline perimetry is recommended [31]. Two case reports of TPM-induced field abnormalities in patients without concomitant ciliochoroidal effusions have been published. In the first case, the use of TPM for migraine prophylaxis was reportedly associated with right incongruous homonymous hemianopia. While in the second case, the patient developed homonymous hemianopia after TPM use for 12 weeks. Both cases observed partial recovery following drug discontinuation [32-33]. Visual field defects associated with TPM use include heteronymous and homonymous hemianopia, tunnel vision or peripheral vision loss, and scotoma [34]. The precise mechanism of drug-induced visual field defects is not well-understood. One published study assessed the effects of chronic TPM administration in rabbits. It demonstrated a significant reduction in retinal function. In the inner retina, immunohistochemical alterations due to substantial accumulation of GABA were also observed [35]. The documented morphological alterations and retinal dysfunction infer that TPM usage may cause damage to the retina [36].

Myokymia

Eyelid myokymia is a mild muscle contraction of the eyelid that typically affects only one lid. It more commonly affects the lower eyelid but can also impact the upper eyelid. Patients may notice their eyelids twitching, but others may not notice this movement. Contractions are episodic and self-limited, sometimes lasting seconds to hours or even weeks [37]. Myokymia is linked to stress, anxiety, fatigue, and excessive consumption of caffeine [38]. Medication-induced myokymia is rare. Although, it has been reportedly associated with TPM use. Medrano-Martínez V et al. reported that eight of 140 migraine patients treated with TPM developed eyelid myokymia after beginning treatment. The myokymia in all patients disappeared when the researchers took them off the TPM. However, it was prescribed again, and the myokymia reappeared in all patients. They concluded that eyelid myokymia is an under-reported side effect of TPM use in patients with migraine [39].

Neuro-ophthalmological complications

Oculogyric Crisis

An oculogyric crisis is characterized by a spasmodic movement of the eyeballs into a fixed position due to a reaction to a medication or any other medical issue. In addition to a deviation of the eyes, the most commonly reported abnormalities include lateral and backward neck flexion, ocular pain, tongue protrusion, and a widely opened mouth. The condition might be accompanied by excruciatingly painful jaw spasms that may result in tooth breakage [40]. It is found to be associated with approximately 365 drugs. Only two cases of TPM-induced oculogyric crisis have been documented, both of which happened in 2017. One patient was male, the other female. Both were between the ages of 50 and 59. They were reportedly taking pyridoxine, too [41]. Immediate treatment consists of IV benzatropine or procyclidine. They are typically effective within five minutes, though they may take up to 30 minutes to become fully effective. Additional procyclidine doses may be required after 20 minutes. TPM should be ceased. A total of 25 mg of diphenhydramine can also be used to treat this condition [40].

Alice in Wonderland Syndrome (AIWS)

AIWS describes a set of symptoms involving the alteration of perception, usually involving body image. It can also apply to other objects, mostly at night rather than during the day. The patient incorrectly observes body parts, interpreting them being larger than they are. Often, the head and hands are seen as disproportionately large. The patient may also perceive time as moving faster or slower than in reality. This condition is most often associated with migraine headaches [42]. Two studies have indicated that AIWS may be triggered by TPM, separately from the patient's history of migraine. In 2006, Evans RW described a case of a patient who developed AIWS approximately one week after starting TPM for migraines. The patient had complete resolution of symptoms within one month of stopping the medication [43]. In 2009, Jürgens TP et al. described the case of a 17-year-old girl who experienced AIWS while on TPM for migraines. She had a complete resolution of her symptoms when the medication was discontinued but experienced them again when she was re-administered with the medication [44].

Palinopsia

Palinopsia refers to a group of visual symptoms in which an image's persistence or recurrence occurs. It differs from a physiologic after image, a benign response in which an optical image briefly persists after it is taken away. Palinopsia images may continue after an image is removed or recur after a time interval. While after images are negative images, appearing in complementary colors to the original image, palinopsia images are positive images, appearing in the original image's colors [45]. Several studies have linked TPM use with palinopsia. Most recently, Yun SH et al. presented a case series of nine patients who experienced palinopsia while taking TPM. Most patients experienced complete resolution of the palinopsia after ceasing the medication. The researchers suggested that TPM is similar to other medicines that can cause palinopsia and may inhibit neural activity. They further stated that this condition might be more common in people with a slower baseline of visual processing [46].

Diplopia and Nystagmus

Recently Kocamaz M and Karadag O reported the case of a 24-year-old woman who developed a sudden drop of vision in both eyes and diplopia on the second day of examination. Her medical history revealed that she started to use TPM 12 days earlier. Her best corrected visual acuity was 20/25 in the right eye with -5.50 spherical refractive correction and 20/20 in the left eye with -6.25 spherical refractive correction. The patient's clinical condition was considered to be related to the drug, and TPM was discontinued immediately. However, her clinical condition improved rapidly without treatment, and she experienced four months of photosensitivity [47]. According to this study, diplopia and nystagmus are reported in 14-15% of individuals receiving high TPM doses [47].

Increased Central Corneal Thickness

Kerimoglu H et al. presented a patient with a TPM-induced myopic shift (TiMS) with increased central corneal thickness estimated at 543 µm for the right eye and 561 µm for the left eye. Consequently, the medication was discontinued. The patient's cornea gradually shrank in thickness over the next three weeks, reaching consecutively 528 µm and 536 µm. The researchers hypothesized that TPM's prostaglandin-mediated effect and its weak carbonic anhydrase inhibitor activity might have caused this phenomenon [48].

Scleritis

All research studies mentioning the association of scleritis with TPM refer to a 2004 study conducted by Fraunfelder FW et al. This study notes four patients who experienced scleritis while on TPM [19].

Trichomegaly

Trichomegaly, or hypertrichosis of the eyelashes, is defined by increased length, thickness, stiffness, curling, and pigmentation of existing eyelashes. Trichomegaly can be congenital or acquired secondary to medications or chronic illnesses. A case study by Santmyire-Rosenberger BR et al. discusses a 25-year-old bipolar patient who experienced trichomegaly while taking TPM. One month after cessation of therapy, the trichomegaly subsided [49].

Table 1 shows topiramate-related ophthalmic side effects reported in different research studies.

**Table 1:** Topiramate-related ophthalmic side effects reported in different research studies. AACG: Acute angle-closure glaucoma; TPM: Topiramate; TiACG: Topiramate-induced angle-closure glaucoma; OCT: Optical coherence tomography; IOP: Intraocular pressure; AIWS: Alice in wonderland syndrome.

Study	Outcomes
Banta JT et al. (2001) [13]	A 51-year-old patient developed ciliary body edema with idiosyncratic ciliochoroidal detachment resulting in lens thickening, lens-iris diaphragm’s anterior displacement, and AACG.
Rhee DJ et al. (2001) [14]	A 43-year-old woman experienced mild frontal headache, ciliary body swelling, acute bilateral myopia, and ACG.
Foroozan R and Buono LM (2003) [32]	A migraineur woman, aged 32, experienced right incongruous homonymous hemianopia.
Craig JE et al. (2004) [15]	TPM-associated ciliochoroidal effusion, anterior chamber shallowing, lens-iris diaphragm’s forward displacement resulting in acute myopia was reported in two epileptic women, aged 25 and 45. One patient experienced ACG too.
Santmyire-Rosenberger BR and Albert M (2005) [49]	After taking TPM for bipolar disorder, a 25-year-old Hispanic female patient developed acquired trichomegaly besides mild hypertrichosis of her forearms on both sides.
Rhee DJ et al. (2006) [17]	Combination therapy of mannitol and methylprednisolone rapidly improve severe TiACG that is linked to extremely high IOP. The TiACG may include elements of inflammation.
Asensio-Sánchez VM et al. (2006) [33]	A generalized seizures patient, aged 16, suffered from TPM-associated homonymous hemianopia and a 24-year-old epileptic woman experienced maculopathy in both eyes.
Kjellström S et al. (2006) [35]	6 rabbits experienced retinal dysfunction and immunohistological changes, including severe GABA accumulation in inner retina.
Evans RW (2006) [43]	Palinopsia has been reported in two migraineurs. A third patient developed the AIWS after TPM use for migraine treatment.
Edell E et al. (2007) [20]	A -3.00 myopic Caucasian woman, aged 28, experienced hours-long blurred vision, ciliary body edema, and lens-iris diaphragm’s forward displacement in both eyes. This is the largest reported myopic shift case induced by TPM.
Mandal A et al. (2008) [36]	TPM caused bilateral arcuate field defects and superior quadrantanopia in two separate cases.
Kerimoglu H et al. (2009) [48]	A 29-year-old female migraineur experienced blurred vision along with acute myopia, shallow anterior chamber, central corneal thickening, and lens-iris diaphragm’s forward displacement.
Natesh S et al. (2010) [21]	A 23-year-old man was diagnosed with angle closure, macular striae, and acute myopia. This is the first report to document the development of striae following low TPM doses and their diminution.
Jabbarpoor Bonyadi MH et al. (2011) [27]	A 40-year-old migraineur woman experienced bilateral eye pain and blurred vision accompanied by high IOP and a shallow anterior chamber. Following TPM discontinuation, the patient experienced bilateral anterior uveitis with hypopyon and posterior synechiae.
van Issum C et al. (2011) [11]	The first report of anterior segment OCT revealed bilateral TiACG accompanied by acute myopia, elevated IOP, ciliochoroidal detachments, ciliary body’s anterior rotation in a patient. Pilocarpine exacerbated the condition.
Jürgens TP et al. (2011) [44]	A 17-year-old female migraineur, without aura, experienced intermittent AIWS, alopecia and paresthesia in her lips, toes, and fingertips. TPM was linked to her mood swings, aggressive behavior, and depressive symptoms. Furthermore, she experienced intermittent nocturnal body image distortions following sleep delay. Another female migraineur, aged 31, experienced drowsiness, anorexia, sedation, paresthesia, abdominal pain, and difficulty in concentrating.
Abtahi MA et al. (2012) [9]	A systemic review of 74 studies. 65 of them were small-scale observational studies stating TPM-induced ocular adverse effects in 84 patients. Among them, 66 patients developed ciliochoroidal effusion syndrome, 49 suffered from ACG, and 17 experienced myopic shift. Rare side effects including ophthalmic inflammatory reactions, massive choroidal effusion, visual field defects, neuro-ophthalmologic complications and effects on cornea, retina, and sclera were also studied.
Cole KL et al. (2012) [12]	Bilateral AACG developed in a migraine patient after 2 days of TPM use.
Gualtieri W and Janula J (2013) [25]	The first report of a pure TPM maculopathy with cellophane-like reflex and macular striae that was not accompanied by myopia and/or acute glaucoma. A 22-year-old female migraineur experienced bilateral visual acuity deterioration accompanied by choroidal layers plicae and congruent retinal folds.
Fraunfelder FW et al. (2013) [19]	There were 86 reports of acute glaucoma (3 unilateral and 83 bilateral), 17 acute myopia cases, 9 suprachoroidal effusions cases, 3 periorbital edema reports, and 4 scleritis cases. For secondary AACG, peripheral iridectomy is ineffectual.
Pikkel YY (2014) [30]	An obese and binge-eating patient, aged 54, developed panuveitis and AACG in both eyes.
Sears N et al. (2015) [22]	A 7-year-old male with sudden-onset myopic shift and symmetric retinal macular striae along with anterior chamber shallowing, attached hyaloid, and ILM corrugation.
Medrano-Martínez V et al. (2015) [39]	8 people with migraine developed eyelid myokymia.
Yun SH et al. (2015) [46]	Palinopsia developed in 13 female patients. Comorbidities included bulimia nervosa, migraine, and idiopathic intracranial hypertension. The majority of the patients had exacerbated visual disturbance late at night or in the early morning.
DaCosta J and Younis S (2016) [24]	An IgG4-related disease patient, aged 32, developed TPM-induced reversible maculopathy and reduced vision along with cystoid macula edema and anterior uveitis.
Goldberg JL et al. (2016) [28]	Uveitis rarely, if ever, occurs with TPM use. There is no conclusive evidence of a duration or dose-dependent affiliation between TPM consumption and uveitis.
Haque S et al. (2016) [31]	A 34-year-old woman with migraine developed inferior visual field defects that were incongruous in both eyes. TPM usage is also associated with visual scotomas caused by maculopathy and retinal damage.
Khalkhali M (2016) [38]	After using TPM to treat her binge eating disorder, a 47-year-old woman developed persistent eyelid myokymia.
Rosenberg K et al. (2017) [26]	TPM-associated macular neurosensory retinal detachment in two female patients was reported for the first time.
Lan YW and Hsieh JW (2018) [1]	TPM-associated bilateral myopic shift and AACG because of ciliochoroidal effusion that leads to anterior chamber shallowing and lens thickening in two middle-aged women. The former was caused primarily by the lens-iris diaphragm's anterior displacement.
Mahendradas P et al. (2018) [29]	A 36-year-old female migraineur experienced redness, photophobia, pain, and a sudden decrease in vision. She was suffering from bilateral AACG and severe panuveitis with pigments and fibrinous exudate in the left eye’s anterior chamber. Her choroidal thickness decreased and the fibrin material from the left eye’s anterior chamber gradually disappeared.
Quist TS et al. (2019) [18]	In the first case, a 29-year-old female experienced bilateral blurred vision, nearsightedness/myopia, headache, faint halos around bright lights, and severe brow pain accompanied by a skin rash. She had TPM-associated AACG. In the second case, a 29-year-old male developed bilateral reduced/blurry/cloudy vision, headache, and saw halos around bright lights along with a persistent headache, ocular fullness, and sharp eye pain in both eyes.
Muñoz Morales A et al. (2019) [23]	Neurosensory detachments developed in a 36-year-old female suffering from idiopathic intracranial hypertension. TPM can instigate macular and retinal neurosensory detachment.
Kocamaz M and Karadag O (2019) [47]	A 24-year-old epileptic female experienced bilateral vision loss along with diplopia, acute myopia, and photosensitivity.

## Conclusions

Over the years, there has been an increase in TPM use, accompanied by an expanded list of indications, both on and off-label. Many more people are taking this medication in monotherapy or combination with other medicines. Millions of prescriptions are being written per year. Consequently, this increases the number of patients at risk of ocular complications. For patients referred with the aforementioned ocular issues and a history of TPM usage, ophthalmologists should consider refraction tests, thorough fundus examinations, visual field evaluations, and IOP measurement. It is crucial to understand that TPM side effects can be confused with migraine-related pain or a visual aura. As a result of such a misdiagnosis, the attending physician may increase a patient's TPM dosage by mistake. Health-care providers, as well as patients, need to be aware and educated about these issues. Urgent treatment may be necessary to save a patient's sight. Obtaining a baseline ophthalmologic exam before starting TPM is a prudent idea.
